# Field-training in young two-year-old thoroughbreds: investigating cardiorespiratory adaptations and the presence of exercise induced pulmonary hemorrhage

**DOI:** 10.1186/s12917-024-03997-x

**Published:** 2024-04-26

**Authors:** Shannon Massie, Warwick Bayly, Hajime Ohmura, Yuji Takahashi, Kazutaka Mukai, Renaud Léguillette

**Affiliations:** 1 Faculty of Veterinary Medicine, University of Calgary, 3330 Hospital Drive NW, Calgary, Alberta T2N 4Z6 Canada; 2 College of Veterinary Medicine, Washington State University, Grimes Way, Pullman, WA 99164 USA; 3 Equine Research Institute, Japan Racing Association, 1400-4, Shiba, Shimotsuke-shi, Tochigi, 329-0412 Japan

**Keywords:** Heart rate, Ventilation, EIPH, Lactate

## Abstract

**Background:**

Comparatively little is known regarding the initial cardiorespiratory response of young racehorses to training. The objectives were to compare physiological parameters before and after introductory training and determine whether young Thoroughbreds show endoscopic signs of exercise-induced pulmonary hemorrhage (EIPH). Ten Thoroughbreds (20–23 months) underwent 12-weeks of introductory training, including weekly speed sessions. Two 600 m high-speed exercise tests (HSET) were performed following weeks 4 and 12 while wearing a validated ergospirometry facemask. Peak oxygen consumption (V̇O_2_pk) and ventilatory parameters (tidal volume, V_T_; peak inspiratory and expiratory flow, PkV̇_I_, PkV̇_E_; respiratory frequency, Rf; minute ventilation, V̇E) were measured. The ventilatory equivalent of oxygen (V̇E/V̇O_2_) and the aerobic and anaerobic contributions to energy production were calculated. Maximal heart rate (HR_max_) and HR at maximal speed (HR_Vmax_) were determined. Post-exercise hematocrit, plasma ammonia and blood lactate were measured. Evidence of EIPH was investigated via tracheobronchoscopy post-exercise. Results were compared (paired t-test, *P* < 0.05).

**Results:**

Horses were faster following training (*P* < 0.001) and V̇O_2_pk increased 28 ml/(kg total mass.min) (28 ± 16%; *P* < 0.001). Ventilatory (V̇E, *P* = 0.0015; Rf, *P* < 0.001; PkV̇_I_, *P* < 0.001; PkV̇_E_, *P* < 0.001) and cardiovascular parameters (HR_max_, *P* = 0.03; HR_Vmax_, *P* = 0.04) increased. The increase in V̇E was due to greater Rf, but not V_T_. V̇E/V̇O_2_ was lower (26 ± 3.6 vs 23 ± 3.7; *P* = 0.02), indicating improved ventilatory efficiency. Anaerobic contribution to total energy production increased from 15.6 ± 6.1% to 18.5 ± 6.3% (*P* = 0.02). Post-exercise hematocrit (*P* < 0.001), plasma ammonia (*P* = 0.03) and blood lactate (*P* = 0.001) increased following training. Horses showed no signs of EIPH.

**Conclusions:**

Young two-year-old Thoroughbreds responded well to introductory training without developing tracheobronchoscopic evidence of EIPH.

## Introduction

The cardiorespiratory and metabolic effects of treadmill exercise training programs are well described in Thoroughbred (TB) and Standardbred (SB) horses [1–10]; however, comparatively little is known about the responses of young TB racehorses to their initial racetrack training program [11–16]. Although the physiological effects of training, including maximal aerobic capacity (V^˙^O_2max_), have been examined during standardized exercise tests on high-speed treadmills, the translation to field conditions is limited as treadmill tests do not encompass the external stressors of field conditions, including the presence and nuances of a rider.

There have been several attempts to measure V^˙^O_2_ in the field under saddle [17–24]. Despite success at lower intensity exercise, maximal efforts have been hindered by mask design, specifically the need to control horses and minimize the impedance of airflow through the mask. Until recently, field testing has relied on speed, heart rate (HR), electrocardiograms (ECG) and hematological variables to determine the effects of training. The physiological demands of exercise, including spirometry and ventilatory parameters, remain poorly defined in maximal exercise field conditions.

It is well known that TBs have a high prevalence of exercise-induced pulmonary hemorrhage (EIPH) [25]. Post-race tracheobronchoscopies have found that approximately 75% of TBs show evidence of EIPH and that the prevalence in at least one assessment increases to 95% with repeated examinations [26]. Current research suggests that EIPH is a consequence of the physiologic responses to high intensity exercise, though there is a paucity of data supporting when EIPH first occurs in the early career of racehorses. Microscopic lesions associated with EIPH have been found on post-mortem in horses under 2 years of age that had been undergoing race training [27]. While it has been postulated that EIPH is associated with age [28, 29], confounding factors, such as lifetime race starts and the cumulative volume of high intensity exercise, have been shown to be more important than individual risk factors, such as age or sex [30]. The number of days between racing, ambient temperatures and weight carried have also been shown to be associated with EIPH scores [25]. A recent study in young TBs revealed race location and some track surfaces (dirt and turf) were associated with greater prevalence and severity of EIPH, though further investigation is warranted, as the reasons for this remain unclear [31].

Most studies in young TB horses have focused on skeletal and muscular adaptations to training and racing. Few have examined the metabolic response during the early stages of training, and to our knowledge, none have assessed the endoscopic evidence of EIPH. It remains unclear as to how introductory training might (or might not) affect the risk of developing EIPH in young racehorses. Additionally, no studies have described the ventilatory responses to field-tests in this population of horses. The present study aimed to provide a more comprehensive understanding of how field-training impacts the development of aerobic capacity, specifically ventilation, to provide greater insight into responses to initial training and athletic potential of young Thoroughbred racehorses. The objectives were: (1) to compare the maximal cardiorespiratory and metabolic responses in young two-year-old Thoroughbreds before and after a typical track-based introductory training protocol and, (2) to determine whether young two-year-old Thoroughbreds show endoscopic signs of EIPH following introductory training sessions. It was hypothesized that horses would exhibit improvements in cardiorespiratory parameters and show endoscopic signs of EIPH following introductory racetrack training.

## Methods

### Study design

This was a prospective study on an available cohort of young Thoroughbred racehorses undergoing introductory training. The study took place in Miyazaki, Japan and was approved by the Animal Welfare and Ethics Committee of the Japan Racing Association (Approval number, 19–2; Approval date, December 28, 2018). All horses were owned by the Japan Racing Association and had previously completed basic under-saddle (walk, trot, slow canter) training. Informed consent was obtained from all horse owners. All methods were performed in accordance with the relevant guidelines and regulations. Following the study, horses continued with their regular training protocol.

### Horses

Ten clinically healthy Thoroughbred racehorses (four colts, six fillies) from the same training barn (aged 20–23 months at the start of training) underwent a 12-week introductory track-based training program. Horses were stabled individually and fed a standardized diet (mixture of Timothy hay, lucerne hay, oats, green Italian ryegrass, and compounded feed) three times daily with ad libitum access to water. Horses were exercised daily each morning and had individual (for the colts) and group turnout (for the fillies) in large paddocks for the remainder of the afternoon. They were regularly assessed for lameness by a veterinarian.

### Training protocol

Horses were exercised daily for 60 minutes in a circular mechanical walker and exercised under saddle 6 days a week on a 1600 m oil-sand track. Training included one high-intensity speed session per week, progressing from one furlong in less than 18 seconds (approx. 11 m/s) to four furlongs in under 56 seconds (approx.14 m/s) (Table 1). Total daily training equaled approximately 6700–6900 m and consisted of walking (2500 m), trotting (1200 m) and cantering (3000–3200 m).

**Table 1:** Overview of the introductory track-based training schedule. Ten young two-year-old Thoroughbreds exercised under saddle 6 days a week, including a once weekly high-speed sprint training session (time to complete each furlong, F, is included). A high-speed standardized exercise test (HSET) was performed at the end of week-4 (early-training) and week-12 (late-training) for data collection

Date	Daily Distance (m)				Once weekly speed training (time/furlong)	High Speed Exercise Test (HSET)
	Walk	Trot	Canter	Total		
January	2500	1200	3000 ~ 3200	6700 ~ 6900	18 sec/1F (11.1 m/s)	
					36 sec/2F (11.1 m/s)	End of week 4
February					15 sec/1F (13.3 m/s)	
					45 sec/3F (13.3 m/s)	
March					14 sec/1F (14.3 m/s)	
					56 sec/4F (14.3 m/s)	End of week 12

### High-speed exercise tests

Horses completed two high-speed exercise tests (HSET); one at the end of week 4 (“early-training”) and one at the end of week 12 (“late-training”). Horses and jockeys (including tack) were weighed on a digital scale (Hw-400, Belltech Co., Ltd., Osaka, Japan) prior to exercise. After completing a walk, trot, canter warm-up, horses were fitted with an ergospirometry facemask (additional details below). Each horse completed a 1000 m high-speed sprint, progressively increasing to maximal speed which was sustained for the last 600 m. Four jockeys were randomly assigned to each horse to minimize the effects of a rider and were instructed to push each horse to run as fast as possible. No other horses were present on the track to minimize the effects of external environmental factors.

### Heart rate and speed

Horses were fitted with a dual polar heart rate and GPS monitor (RC3 GPS, Polar, Kempele, Finland) secured under their saddle, to measure maximal heart rate (HR_max,_ beats per minute, bpm) and speed (m/s). Additionally, the HR at maximal speed (HR_Vmax_) was recorded, as well as the speed (m/s) and time (s) to complete each furlong. Data was imported into an Excel spreadsheet for analysis.

### Oxygen consumption and ventilatory parameters

Peak oxygen consumption (V̇O_2_pk; ml/(kg.min) and L/min), defined as the peak oxygen consumption reached during the final stage of the high-speed exercise test, was measured using a portable ergospirometry facemask, previously validated for maximal intensity field-exercise in horses [32]. Data was reported relative to the horse’s (H) weight (ml/(kg H.min)) as well as the combined weight of the horse and jockey, including tack (HJ; ml/(kg HJ.min)). Horses had not previously worn the facemask, which fit over their regular bridle and ensured the jockeys had complete control of the horse. Dead space was minimized by adjusting the fit of the mask to each horse. Jockeys wore a small backpack that housed a battery-operated metabolic analyzer and computer tablet. Data was recorded on the tablet and wirelessly synced with a nearby laptop. Results were displayed using customized software. Oxygen sensors were calibrated before and after each run using room air, accounting for changes in ambient temperature, barometric pressure, and humidity (Barometer Plus, Ngo Na, Apple App Store), and a certified medical grade gas (16.00% O_2_). In addition to V̇O_2_pk, breath-by-breath spirometry parameters were measured, including minute ventilation (V̇E, L/min), respiratory frequency (Rf, breaths per minute, bpm), peak inspiratory flow (PkV̇_I,_ L/s), peak expiratory flow (PkV̇_E,_ L/s) and tidal volume (V_T,_ L). The ventilatory equivalent for V̇E/V̇O_2_pk was determined as a measure of ventilatory efficiency. Aerobic capacity (corrected to standard temperature and pressure, dry; STPD) is presented relative to horse’s own weight (ml/(kg H.min)) and the total weight (horse + jockey) moved through space during the exercise test (ml/(kg HJ.min)); this encompassed the weight of the jockey and tack. Data was averaged over the final 15 seconds of the maximal sprint test.

### Blood samples

Blood samples (4 ml) were drawn from the left jugular vein into EDTA (VP-NA052K, Terumo Corp., Tokyo, Japan) and lithium heparin tubes (VP-H100K, Terumo Corp., Tokyo, Japan) before and immediately following exercise. Blood was immediately transferred from the EDTA tube into a capillary tube and spun in a microhematocrit centrifuge. Hematocrit (%) was determined visually and a small amount of whole blood (< 20 μL) from the EDTA tubes was applied to test strips and inserted into handheld analyzers measuring ammonia (Ammonia Test Kit II, The PocketChem™ BA PA-4140, Arkray Inc., Kyoto, Japan) and from the heparinized tubes to measure lactate (Lactate Pro2, Arkray Inc., Kyoto, Japan).

### Aerobic and anaerobic contribution

Relative aerobic and anaerobic contributions to energy production were calculated using V̇O_2_ and delta blood lactate measurements as previously described [33].

### Post-exercise EIPH

Post-exercise EIPH was investigated via tracheobronchoscopy 30 minutes after the high-speed exercise tests. A 1.5-m long high-definition videoendoscope (VQ Type 8143B, Olympus Corp., Tokyo, Japan) was inserted into the horse’s left nostril with the assistance of a twitch (no sedation was used). The scope was inserted to the level of the carina. All examinations were recorded, and the presence of blood and tracheal mucus was scored immediately on site using a validated grading system [34, 35]. Videos were later re-examined by two experienced clinicians to ensure scoring agreement.

### Statistics

Normality was confirmed using a Shapiro-Wilk test and early-training and late-training measures were compared using a paired t-test (GraphPad Prism 9). The effects of training and sex on running times were assessed using a two-way repeated measures ANOVA. Results are presented as mean ± SD unless otherwise noted. Significance was set for *P* < 0.05.

## Results

Environmental conditions during the high-speed peak exercise tests showed similar humidity *(P* = 0.90) and barometric pressures (*P* > 0.99) with a warmer ambient temperature (*P* = 0.008) on the days of the late-training (15.2 °C ± 1.7) than the early-training (10.8 °C ± 2.1) exercise tests.

### Horses

All 10 horses completed the introductory training program and both HSET. A significant increase (*P* < 0.001) in body weight was observed from early-training (457.9 ± 26.7 kg) to late-training (475.0 ± 23.2 kg). The same jockeys were used in both HSETs and no differences in weight were observed (early-training: 75.7 ± 15.1 kg; late-training: 73.6 ± 16.4 kg).

### Heart rate and speed

Significant increases in peak speed were observed from early-training to late-training (13.3 ± 0.5 m/s; 16.0 ± 0.6 m/s; *P* < 0.001) and as a result, horses’ 600 m times were faster. The time to complete the final 400 m decreased by 16% (from 30.9 ± 1.2 sec to 26.1 ± 0.9 sec, *P* < 0.001). Maximal heart rate and HR_Vmax_ both increased significantly from early-training (221 ± 8 bpm; 219 ± 9 bpm) to late-training (226 ± 7 bpm, *P* = 0.03; 223 ± 8 bpm, *P* = 0.04). In both the early- and late-training HSET, HR remained above 200 bpm for two consecutive minutes, confirming that horses were performing at or close to maximal intensity exercise. The time in training influenced running time, with horses running each 200 m segment faster during the late-training HSET. There was no effect of sex on any results (*P* = 0.92). Time to complete each furlong is shown in Table 2.

**Table 2:** Data from 10 young two-year-old Thoroughbred horses undergoing introductory track-based training. Mean maximal heart rate (HR_max_, bpm), peak speed (V_max_, m/s) and maximal HR at maximal speed (HR_Vmax_) and time to complete each furlong (sec) during the early-training (week 4) HSET and late-training (week 12) HSET. Data presented as mean ± SD

	Early-Training	Late-Training	*P* Value
HR_max_ (bpm)	221.0 ± 7.9	225.8 ± 6.9	0.030
V_max_ (m/s)	13.3 ± 0.5	16.0 ± 0.6	< 0.001
HR_Vmax_ (bpm)	219.3 ± 8.5	222.7 ± 7.8	0.045
1st furlong (s)	20.2 ± 2.3	16.9 ± 1.3	< 0.001
2nd furlong (s)	18.6 ± 2.0	16.0 ± 0.9	0.0016
3rd furlong (s)	17.0 ± 1.2	15.0 ± 1.3	0.007
4th furlong (s)	15.5 ± 0.7	13.4 ± 0.6	< 0.001
5th furlong (s)	15.3 ± 0.5	12.7 ± 0.5	< 0.001
Final two furlongs (avg)	30.9 ± 1.2	26.1 ± 0.9	< 0.001

### Oxygen consumption and ventilatory parameters

**Fig. 1:**
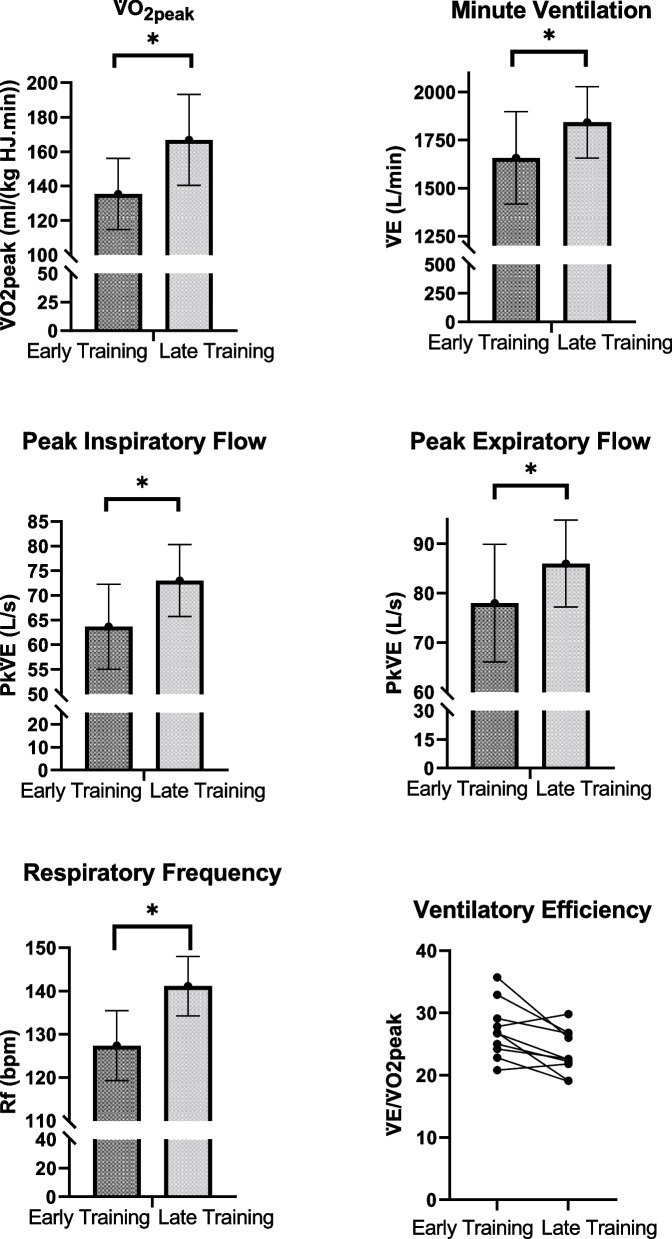
Ergospirometry parameters measured during HSET in early-training (week-4) and late-training (week-12) in 10 young two-year-old racing Thoroughbreds. Parameters include absolute peak aerobic capacity (V̇O_2_pk, L/min), relative (to horse) peak aerobic capacity (V̇O_2_pk, ml/kg H.min), total mass (horse and jockey) peak aerobic capacity (V̇O_2_pk, ml/kg HJ.min), peak inspiratory flow (PkV̇_I_, L/s), peak expiratory flow (PkV̇_E_, L/s), peak inspiratory flow (PkV̇_E_, L/s), respiratory frequency (Rf, bpm), minute ventilation (V̇E, L/min) and ventilation efficiency (V̇E/ V̇O_2_pk). * Indicates a statistical difference (*P* < 0.05)

Absolute (L/min) and relative (ml/(kg H.min)) V̇O_2_pk increased by 28 and 23%, respectively, from the early-training to late-training HSET. Following the 8 weeks of training, mean relative V̇O_2_pk increased from 116.3 ± 18.1 to 144.7 ± 23.6 ml/(kg HJ.min) (*P* < 0.001) and 135.3 ± 20.7 to 166.9 ± 26.4 ml/(kg H.min) (*P* < 0.001), while absolute V̇O_2_pk increased from 61.8 ± 9.6 to 79.3 ± 13.1 L/min (*P* < 0.001). Significant increases were observed in V̇E (early-training: 1657 ± 241 L/min; late-training: 1843 ± 186 L/min; *P* = 0.0015), Rf (early-training: 127 ± 8 bpm; late-training: 141 ± 7; *P* < 0.001), PkV̇_I_ (early-training: 63.7 ± 8.6 L/s; late-training: 73.0 ± 7.3 L/s; *P* < 0.001) and PkV̇_E_ (early-training: 79.3 ± 11.9 L/s; late-training: 86.6 ± 8.8 L/s; *P* < 0.001) but not in V_T_ (early-training: 13.2 ± 2.2; late-training: 13.2 ± 1.7; *P* = 0.49). Results are shown in Fig. 1. The V̇E/V̇O_2_ decreased with training (early-training: 26 ± 3.6, late-training: 23 ± 3.7; *P* = 0.02).

### Aerobic and anaerobic contribution

Aerobic contribution to total energy production decreased from 84.4 ± 6.1% to 81.5 ± 6.3% (*P =* 0.02) while anaerobic contribution subsequently increased from 15.6 ± 6.1% to 18.5 ± 6.3% (*P =* 0.02).

### Blood tests

Post-exercise hematocrit, plasma ammonia and whole blood lactate concentrations increased significantly from early-training to late-training (Table 3).

**Table 3:** Data from 10 young two-year-old Thoroughbred horses undergoing introductory track-based training. Post-exercise whole blood hematocrit (HCt, %), plasma ammonia (NH_3,_ μmol/L) and blood lactate (mmol/L) between the early-training (week 4) HSET and late-training (week 12) HSET. Values presented as mean ± SD

*n* = 10	Early-Training	Late-Training	% Change	*P* Value
Post-exercise HCt (%)	55.4 ± 2.4	59.7 ± 2.4	+ 7.8%	< 0.001
Post-exercise NH_3_ (μmol/L)	59.0 ± 15.4	76.3 ± 32.4	+ 29.3%	0.03
Post-exercise blood lactate (mmol/L)	9.1 ± 3.5	13.5 ± 5.0	+ 48.4%	0.001

### Post-exercise EIPH

Horses showed no tracheobronchoscopic signs of EIPH following either the early-training or late-training HSET. A small amount of tracheal mucus (mean score 1.1 ± 0.7) was observed in almost all horses (*n* = 9) following both HSET.

## Discussion

Young two-year old Thoroughbreds responded well to introductory track-based training, as evidenced by improvements in multiple cardiorespiratory parameters between the early- and late-training HSET. Additionally, no horses showed any tracheal signs of EIPH. Horses exhibited improvements in peak running speeds (+ 20%) and the time to complete two furlongs (15% faster). This was likely related to the cardiovascular and muscular adaptations of training [36], including metabolic adaptations that delay the onset of fatigue [37]. Male horses have previously been shown to reach higher peak speeds than female horses [38], however, like Fonseca et al. [39], we found no differences in peak speeds between fillies and colts. Variations in speed may be related to differences in lean muscle mass amongst young racehorses. Although measuring body composition was beyond the scope of this study, a significant increase in body weight was observed between the early- and late-training HSET. This was presumably due to increases in lean muscle mass, as fat-free mass has been shown to increase due to the combination of training and natural growth and maturation [40]. Increases in stride frequency at a given speed have also been observed in young TBs undergoing training, although interpretation of the data remains unclear [37].

In human athletes, maximal aerobic capacity is considered one of the most important factors for predicting an individual’s health and athletic potential and is routinely used to assess aerobic performance capacity [41]. Maximal oxygen consumption is associated with a plateau in V̇O_2,_ despite a continual increase in workload. As this is very difficult to demonstrate under field conditions, peak V̇O_2_ (V̇O_2peak_) is often reported. Previous studies in TB racehorses have shown significant improvements in V^˙^O_2_ parameters following treadmill training [6, 7, 9, 10, 42] and conventional track-based training [13–15, 43]. Untrained yearlings and two-year old TBs have been shown to have mean baseline V̇O_2_pk scores measuring 128 ml/(kg.min) and 140 ml/(kg.min) respectively [12]. It has been suggested that the early “breaking” phase of training results in the most significant increases in V̇O_2_pk in young TBs due to rapid changes in cardiorespiratory parameters. One study found a 21% increase in absolute V^˙^O_2_ (57 to 69 L/min) and 13% increase in relative V^˙^O_2_ (134 to 151 ml/(kg.min)) in young TBs undergoing low intensity training during the early phase of training [14]. Higher baseline V̇O_2_pk scores (154 ml/(kg.min)) have been observed in two-year-old TBs that have undergone the early stages of training [13]. The lack of subsequent improvement with low intensity training in two-year-old TBs further supports that horses experience large adaptations in the early phases of training and thus require progressively higher intensity exercise to maximize aerobic capacity.

Drawing meaningful comparisons between studies is challenging due to a myriad of differences including training status, exercise type, intensity and duration, and gas collection systems used (open flow, differential pressure transducers and ultrasonic flow transducers). Until recently [32], no system had been validated for measuring V^˙^O_2_ in the field under high-intensity exercise. The development of a light weight, portable mask that allows for rider intervention has been shown to provide comparable results to a traditional open-flow gas collection system used on a high-speed treadmill [32]. This novel portable device provides greater insight to the physiological demands and nuances of field-testing, including the presence of a jockey. In the current study, horses had already undergone the early stages of training and had completed 4 weeks of introductory track-based training prior to the first HSET. The mean increases in relative V̇O_2_pk adjusted for total mass (horse, jockey, and tack) was 28 ml/(kg HJ.min) (+ 24%) from early-training (116.3 ml/(kg HJ.min)) to late-training (144.7 ml/(kg JK.min)). Mean jockey weight did not change; however, to account for the increase in mean horse weight, we chose to also examine absolute V̇O_2_pk, which increased by 28% (62 to 79 L/min). This increase indicates that training levels were sufficient to result in cardiorespiratory and muscle improvements. Mass specific V̇O_2_pk scores were comparable to what has previously been reported in young TBs undergoing higher intensity training [13], though it is important to mention that maximal intensity field-testing is in some measures less standardized than high-speed treadmill testing. Although several parameters confirm maximal intensity exercise, accurate tests rely on the encouragement of each jockey and the subjective effort of each horse.

Improvements in aerobic capacity are the result of several central and peripheral training adaptations, primarily cardiac output and an improved oxygen carrying capacity of the blood [44]. Despite a significant increase in maximal heart rate following training, the difference was not clinically relevant as both exercise tests revealed maximal HRs above 220 bpm. Previous studies have already shown that training does not alter maximal heart rate in any age group of horses [45]. Data pertaining to changes in stroke volume (SV) has been mixed, with an early study by Thomas et al. [2] showing improvements in SV following training, while another by Bayly et al. [1] did not. Although incremental treadmill tests have shown no significant changes in SV from resting values [3], larger improvements have been observed following 7 weeks of treadmill training [4]. Echocardiographic data also confirms that relative left ventricle mass and wall thickness increases with training in young Standardbred and National Hunt horses [46, 47], thereby increasing cardiac output. These adaptations have been shown to be positively correlated with racing performance.

Ventilatory parameters were reported in young TBs in the field for the first time. Due to the breath-by-breath design of the current facemask, peak V_T_, and peak inspiratory and expiratory flow, breathing frequency and total ventilation are reported. Increases in V̇E (+ 11%) were due to a higher respiratory frequency (+ 11%), but not V_T_. It has previously been shown that V_T_ does not change with training in mature Thoroughbreds [7]. This is also presumed to be true in young TBs undergoing training. The increase in Rf was likely associated with an increase in stride frequency (and thus faster speeds) due to the tight coupling of locomotion and breathing. Peak inspiratory and expiratory flows also increased, indicating that horses had to generate higher peak negative and positive pressures to inspire and expire the same V_T_ in less time per breath (because Rf is faster).

Age and training status influence the hematologic profile and aerobic capacity of the athletic horse [48]. Haematological changes, including increases in plasma volume, hematocrit, and the number of circulating red blood cells, have been described in young TB horses following races [49] and 4-months of track-based training [50, 51]. These adaptations improve oxygen carrying capacity via increases in hemoglobin concentrations and cardiac output and, together with increased capillarization of skeletal muscle and increased activity of enzymes involved in oxidative phosphorylation, enhance aerobic capacity. This improved delivery and utilization of oxygen by the exercising muscles results in a decrease in the V̇E/V̇O_2_ and is interpreted as reflecting an enhancement in the efficiency of ventilation as observed in this study. The effects of young horses’ initial exposure to training on ventilatory efficiency have not been previously reported and are additional evidence of the positive metabolic responses of these young Thoroughbreds to their training program.

The increase in post-exercise blood lactate concentration was expected following training due to improvements in peak running speeds. Lactate values were similar to those reported by Evans et al. [52] in horses undergoing an 800 m maximal field test; however, were lower than those reported in horses undergoing a 1200 m race [53]. Interestingly, the current study contradicts findings from a similar study whereby TBs underwent a 600 m SET and found lower plasma lactate following 6-weeks of interval training, despite faster times [54]. A large amount of variability exists in lactate values reported in TB yearling training studies [13–16]. This is due to differences in exercise intensity, environments (treadmill vs. track; training vs. competition), methodologies (intravenous catheter vs. jugular sample, whole blood vs. plasma) and the type of analyzers used. The timing of sampling also has a large effect. In the current study, blood was pulled immediately following exercise which may have potentially limited the time for intracellular lactate to diffuse into the blood, thus causing concentrations to be lower. Post-exercise plasma ammonia is a marker of the anaerobic demands of exercise and is considered a sensitive indicator of performance, muscle fatigue and training efficiency [55, 56]. Although in the current study no serial measurements were obtained, a 30% increase in post-exercise plasma ammonia was observed following training, suggesting a rightward shift in the ammonia threshold curve, otherwise representative of a delay in the onset of fatigue and thus enhanced performance. Reference ranges for maximally exercising racehorses in the field are lacking, however we found that values were similar to those obtained in incremental high-speed treadmill studies [57].

The effects of training on the relative contribution of aerobic and anaerobic energy production can be estimated using serial V̇O_2_ measures and delta blood lactate measurements [33]. In the current study, anaerobic contribution was estimated to be 20% higher following training. These findings suggests that training improves the efficiency of enzymatic systems and metabolic adaptations of skeletal muscles to maximal exercise. Higher anaerobic contributions are compatible with the increased blood lactate concentrations [58] and might also indicate a greater intramuscular buffering capacity and ability to withstand the rise in intracellular lactate and unbuffered hydrogen ions (which are responsible for slowing ATP production) [55].

Horses showed no signs of tracheal blood following either peak exercise test, suggesting that EIPH is not prevalent in young TBs undergoing introductory training. A recent retrospective study of 1071 post-race tracheobronchoscopies found that 74% of two-year old TBs had EIPH and more than 90% of horses examined multiple times exhibited EIPH [31]. The authors found associations between severe EIPH (>Grade 3) and reduced racing performance. When corroborated with the current results, these findings suggest that horses begin to bleed sometime between introductory training and racing. In the current study horses only ran maximally for 600 m, compared to the 900–1800 m run in races [31]. Therefore, it remains unclear whether EIPH is related to crossing a training volume threshold or whether it is related to intensity or distance covered. Microscopic EIPH lesions have been observed in young TBs training at speeds above 7.0 m/s [27], further suggesting that intensity plays an important role in the onset of bleeding. However, the TBs in the current study showed no signs of EIPH, despite regularly training at speeds above 11 m/s. Albeit, the majority of the introductory training was low intensity exercise, remaining below 7 m/s. Although the pathophysiology of EIPH remains unclear, from the perspective of the pulmonary capillary stress failure theory, it is possible that the low total volume of weekly speed-training was not enough to drive transmural pressures beyond the failure threshold and cause horses to experience EIPH.

### Limitations

A limited number of horses were investigated and although horses were in training for 16 weeks, no initial baseline or post-training data was obtained. Ideally, a control group would have also been included; however, as horses came from a high calibre breeding facility, they were all enrolled in training.

While the current mask design has been shown to provide accurate and comparable results, the system lacks a carbon dioxide sensor thus limiting the metabolic parameters we can measure. Nevertheless, V̇E/V̇O_2_ provided some useful information about the effects of training on the ventilatory efficiency. The decrease in the V̇E/V̇O_2_ ratio was small but significant, indicating that more oxygen was extracted per liter of inhaled air following training. Although it would have been interesting to measure exercising blood gases to determine how field training contributes to hypoxemia and hypercapnia, this is not easily undertaken in the field.

Although post-race tracheobroncoscopies are generally accepted as a strong diagnostic tool for EIPH, they lack the sensitivity of a bronchoalveolar lavage [59]. Blood may have been present in the lower airways, but not in volumes large enough to show evidence in the trachea. Serial endoscopic examinations could also be utilized to more accurately determine whether horses have developed EIPH.

## Conclusion

Young two-year-old Thoroughbreds became fitter in response to their first exposure to racetrack training, as measured by higher peak speeds and improved aerobic capacity (as measured by a lightweight, portable ergospirometry facemask). Increases in minute ventilation were due to increases in breathing frequency, but not tidal volume, which was likely related to the increased stride frequency associated with faster speeds and locomotor respiratory coupling. Overall, young two-year old Thoroughbreds responded well to introductory training without developing evidence of EIPH.

## Data Availability

The datasets generated and/or analyzed during the current study are not publicly available due to confidentiality but are available from the corresponding author on reasonable request.

## Abbreviations

EIPH: Exercise induced pulmonary hemorrhage
HR: heart rate
HR_max_: Heart rate max
HR_Vmax_: Heart rate at maximal speed
HSET: High speed exercise test
PkV̇_E_: Peak expiratory flow
PkV̇_I_: Peak inspiratory flow
Rf: Respiratory frequency
V̇E: Minute ventilation
V̇E/V̇O_2_: Ventilatory equivalent of oxygen
V̇O_2_pk: Peak oxygen consumption
V_T_: Tidal volume

## References

[1] Bayly WM, Gabel AA, Barr SA (1983). Cardiovascular effects of submaximal aerobic training on a treadmill in Standardbred horses, using a standardized exercise test. Am J Vet Res..

[2] Thomas DP, Fregin GF, Gerber NH, Ailes NB (1983). Effects of training on cardiorespiratory function in the horse. Am J Phys Regul Integr Comp Phys..

[3] Evans DL, Rose RJ (1988). Cardiovascular and respiratory responses in thoroughbred horses during treadmill exercise. J Exp Biol..

[4] Evans DL, Rose RJ (1988). Cardiovascular and respiratory responses to submaximal exercise training in the thoroughbred horse. Pflugers Arch..

[5] Rose RJ, Hendrickson DK, Knight PK (1990). Clinical exercise testing in the normal thoroughbred racehorse. Aust Vet J..

[6] Knight PK, Sinah AK, Rose RJ (1991). Effects of training intensity on maximum oxygen uptake. Equine Exercise Physiol..

[7] Art T, Lekeux P (1993). Training-induced modifications in cardiorespiratory and ventilatory measurements in thoroughbred horses. Equine Vet J..

[8] Tyler CM, Golland LC, Evans DL, Hodgson DR, Rose RJ (1996). Changes in maximum oxygen uptake during prolonged training, overtraining, and detraining in horses. J Appl Physiol..

[9] Hinchcliff KW, Lauderdale MA, Dutson J, Geor RJ, Lacombe VA, Taylor LE (2002). High intensity exercise conditioning increases accumulated oxygen deficit of horses. Equine Vet J..

[10] Bronsart LL, Sides RH, Bayly WM (2009). A comparative study of interval and continuous incremental training in thoroughbreds. Comparat Exercise Physiol..

[11] Harris MR, Morris EA, Seeherman HJ (1990). Evaluation of the effects of a regular training program in two thoroughbred yearlings using an exercise stress test. J Equine Veterin Sci..

[12] Seeherman HJ, Morris EA (1991). Comparison of yearling, two-year-old and adult thoroughbreds using a standardised exercise test. Equine Vet J..

[13] Hiraga A, Kai M, Kubo K, Sugano S (1997). Effects of low intensity exercise during the breaking period on cardiopulmonary function in thoroughbred yearlings. J Equine Sci..

[14] Hiraga A, Kai M, Kubo K, Sugano S (1997). The effect of training intensity on cardiopulmonary function in 2-year-old thoroughbred horses. J Equine Sci..

[15] Ohmura H, Hiraga A, Matsui A, Aida H, Inoue Y, Asai A, Jones JH (2002). Physiological responses of young thoroughbreds during their first year of race training. Equine Vet J..

[16] Ohmura H, Matsui A, Hada T, Jones JH (2013). Physiological responses of young thoroughbred horses to intermittent high-intensity treadmill training. Acta Vet Scand..

[17] Curtis RA, Kusano K, Evans DL, Lovell NH, Hodgson DR (2005). Reliability of cardiorespiratory measurements with a new ergospirometer during intense treadmill exercise in thoroughbred horses. Vet J..

[18] Art T, Duvivier DH, van Erck E, de Moffarts B, Votion D, Bedoret D, Lejeune JP, Lekeux P, Serteyn D (2006). Validation of a portable equine metabolic measurement system. Equine Vet J..

[19] Votion DM, Caudron I, Lejeune J, van der Heyden L, Art T, van Erck E, Serteyn D (2006). New perspective for field measurement of cardiorespiratory parameters in exercising horses. Pferdeheilkunde..

[20] van Erck E, Votion DM, Serteyn D, Art T (2007). Evaluation of oxygen consumption during field exercise tests in Standardbred trotters. Equine Comparat Exercise Physiol..

[21] Leprêtre PM, Metayer N, Giovagnoli G, Pagliei E, Barrey E (2009). Comparison of analyses of respiratory gases made with the K4b2 portable and quark laboratory analysers in horses. Vet Rec..

[22] Cottin F, Metayer N, Goachet AG, Julliand V, Slawinski J, Billat V, Barrey E (2010). Oxygen consumption and gait variables of Arabian endurance horses measured during a field exercise test. Equine Vet J..

[23] Goachet AG, Julliand V (2015). Implementation of field cardio-respiratory measurements to assess energy expenditure in Arabian endurance horses. Animal..

[24] Fortier J, Deley G, Goachet AG, Julliand V (2015). Quantification of the energy expenditure during training exercises in Standardbred trotters. Animal..

[25] Crispe EJ, Secombe CJ, Perera DI, Manderson AA, Turlach BA, Lester GD (2019). Exercise-induced pulmonary haemorrhage in thoroughbred racehorses: a longitudinal study. Equine Vet J..

[26] Birks EK, Shuler KM, Soma LR, Martin BB, Marconato L, Del Piero F, Teleis DC, Schar D, Hessinger AE, Uboh CE (2002). EIPH: postrace endoscopic evaluation of Standardbreds and thoroughbreds. Equine Vet J..

[27] Oikawa M (1999). Exercise-induced haemorrhagic lesions in the dorsocaudal extremities of the caudal lobes of the lungs of young thoroughbred horses. J Comp Pathol..

[28] Takahashi T, Hiraga A, Ohmura H, Kai M, Jones JH (2001). Frequency of and risk factors for epistaxis associated with exercise-induced pulmonary hemorrhage in horses: 251,609 race starts (1992–1997). J Am Vet Med Assoc..

[29] Weideman H, Schoeman SJ, Jordaan GF, Kidd M (2003). Epistaxis related to exercise-induced pulmonary haemorrhage in south African thoroughbreds. J S Afr Vet Assoc..

[30] Hinchcliff KW, Morley PS, Jackson MA, Brown JA, Dredge AF, O'callaghan PA, McCaffrey JP, Slocombe RF, Clarke AF (2010). Risk factors for exercise-induced pulmonary haemorrhage in thoroughbred racehorses. Equine Vet J..

[31] Shoemaker S, Wang Y, Fisher A, Sellon D, Gold J, Bagshaw J, et al. Prevalence and severity of exercise-induced pulmonary hemorrhage in 2-year-old thoroughbred racehorses and its effect on performance. J Vet Intern Med. 2024;38(2):1167–76. 10.1111/jvim.17003 PMC10937470 38363079

[32] Sides RH, Kirkpatrick R, Renner E, Gough K, Katz LM, Evans DL, Bayly WM (2018). Validation of masks for determination of VO2max in horses exercising at high intensity. Equine Vet J..

[33] Bond SL, Greco-Otto P, Sides R, Kwong GP, Léguillette R, Bayly WM (2019). Assessment of two methods to determine the relative contributions of the aerobic and anaerobic energy systems in racehorses. J Appl Physiol..

[34] Hinchcliff KW, Jackson MA, Brown JA, Dredge AF, O'Callaghan PA, McCaffrey JP, Morley PS, Slocombe RF, Clarke AF (2005). Tracheobronchoscopic assessment of exercise-induced pulmonary hemorrhage in horses. Am J Vet Res..

[35] Gerber V, Straub R, Marti E, Hauptman J, Herholz C, King M, Imhof A, Tahon L, Robinson NE (2004). Endoscopic scoring of mucus quantity and quality: observer and horse variance and relationship to inflammation, mucus viscoelasticity and volume. Equine Vet J..

[36] Rivero JLL, Piercy RJ, Hinchcliff KW, Kaneps AJ, Geor RJ (2014). Muscle physiology. Equine sports medicine and surgery.

[37] Parkes RS, Weller R, Pfau T, Witte TH (2019). The effect of training on stride duration in a cohort of two-year-old and three-year-old thoroughbred racehorses. Animals..

[38] Mukai K, Takahashi T, Hada T, Eto D, Kusano K, Yokota S, Hiraga A, Ishida N (2003). Influence of gender and racing performance on heart rates during submaximal exercise in thoroughbred racehorses. J Equine Sci..

[39] Fonseca RG, Kenny DA, Hill EW, Katz LM (2010). The association of various speed indices to training responses in thoroughbred flat racehorses measured with a global positioning and heart rate monitoring system. Equine Vet J..

[40] Fonseca RG, Kenny DA, Hill EW, Katz LM (2013). The relationship between body composition, training and race performance in a group of thoroughbred flat racehorses. Equine Vet J..

[41] Albouaini K, Egred M, Alahmar A, Wright DJ (2007). Cardiopulmonary exercise testing and its application. Postgrad Med J..

[42] Harkins JD, Beadle RE, Kamerling SG (1993). The correlation of running ability and physiological variables in thoroughbred racehorses. Equine Vet J..

[43] Eaton MD, Hodgson DR, Evans DL, Rose RJ (1999). Effects of low-and moderate-intensity training on metabolic responses to exercise in thoroughbreds. Equine Vet J..

[44] Lundby C, Montero D, Joyner M (2017). Biology of VO_2_max: looking under the physiology lamp. Acta Physiol..

[45] Betros CL, McKeever KH, Kearns CF, Malinowski K (2002). Effects of ageing and training on maximal heart rate and VO_2_max. Equine Vet J..

[46] Young LE (1999). Cardiac responses to training in 2-year-old thoroughbreds: an echocardiographic study. Equine Vet J..

[47] Buhl R, Ersbøll AK, Eriksen L, Koch J (2005). Changes over time in echocardiographic measurements in young Standardbred racehorses undergoing training and racing and association with racing performance. J Am Vet Med Assoc..

[48] Bayly WM (1987). The interpretation of clinicopathologic data from the equine athlete. Vet Clin N Am Equine Pract..

[49] Snow DH, Mason DK, Ricketts SW, Douglas TA, Gillespie JR, Robinson NE (1983). Post-race blood biochemistry in thoroughbreds. proceedings of the first international conference on equine exercise physiology august 1982.

[50] Allen BV, Powell DG (1983). Effects of training and time of day of blood sampling on the variation of some common haematological parameters in normal thoroughbred racehorses. Equine Exercise Physiol..

[51] Miglio A, Cappelli K, Capomaccio S, Mecocci S, Silvestrelli M, Antognoni MT (2020). Metabolic and biomolecular changes induced by incremental long-term training in young thoroughbred racehorses during first workout season. Animals..

[52] Evans DL, Harris RC, Snow DH (1993). Correlation of racing performance with blood lactate and heart rate after exercise in thoroughbred horses. Equine Vet J..

[53] Mukai K, Takahashi T, Eto D, Ohmura H, Tsubone H, Hiraga A (2007). Heart rates and blood lactate response in thoroughbred horses during a race. J Equine Sci..

[54] Bayly WM, Grant BD, Pearson RC (1987) Lactate concentrations in thoroughbred horses following maximal exercise under field conditions. In: Gillespie JR, Robinson NE (eds) Proceedings of the second International Conference on Equine Exercise physiology, august 1986. ICEEP publications, Davis, Calif., 1987 pp 426–37.

[55] Harris RT, Dudley GA (1989). Exercise alters the distribution of ammonia and lactate in blood. J Appl Physiol..

[56] Kremer E, Bond S, Bergsma J, Sides R, Léguillette R, Bayly W (2018). Assessing the ammonia response of thoroughbreds to strenuous exercise with a point-of-care analyzer. Comparat Exercise Physiol..

[57] Harris RC, Harris DB, Dunnett M, Harris PA, Jallowfield J, Naylor JRJ (1999). Plasma ammonia and lactate responses using incremental and constant speed exercise test. Equine Vet J..

[58] Bond S, Greco-Otto P, Sides R, Léguillette R, Bayly WM (2019). Assessment of high-intensity over-ground conditioning and simulated racing on aerobic and anaerobic capacities in racehorses. Comparat Exercise Physiol..

[59] Lopez Sanchez CM, Kogan C, Gold JR, Sellon DC, Bayly WM (2020). Relationship between tracheobronchoscopic score and bronchoalveolar lavage red blood cell numbers in the diagnosis of exercise-induced pulmonary hemorrhage in horses. J Vet Intern Med..

